# Estimating disease incidence rates and transition probabilities in elderly patients using multi-state models: a case study in fragility fracture using a Bayesian approach

**DOI:** 10.1186/s12874-023-01859-y

**Published:** 2023-02-14

**Authors:** Fran Llopis-Cardona, Carmen Armero, Gabriel Sanfélix-Gimeno

**Affiliations:** 1 Health Services Research Unit, Foundation for the Promotion of Health and Biomedical Research of Valencia Region (FISABIO), Valencia, Spain; 2 Department of Statistics and Operations Research. Universitat de València, Valencia, Spain; 3 Joint research unit FISABIO-UV for the analysis of biomedical data, Valencia, Spain; 4 Network for Research on Chronicity, Primary Care, and Health Promotion (RICAPPS), Valencia, Spain

**Keywords:** Bayesian inference, Cause-specific hazard models, Cumulative incidence function, Epidemiological data, Illness-death models, Transition probabilities

## Abstract

**Background:**

Multi-state models are complex stochastic models which focus on pathways defined by the temporal and sequential occurrence of numerous events of interest. In particular, the so-called illness-death models are especially useful for studying probabilities associated to diseases whose occurrence competes with other possible diseases, health conditions or death. They can be seen as a generalization of the competing risks models, which are widely used to estimate disease-incidences among populations with a high risk of death, such as elderly or cancer patients. The main advantage of the aforementioned illness-death models is that they allow the treatment of scenarios with non-terminal competing events that may occur sequentially, which competing risks models fail to do.

**Methods:**

We propose an illness-death model using Cox proportional hazards models with Weibull baseline hazard functions, and applied the model to a study of recurrent hip fracture. Data came from the PREV2FO cohort and included 34491 patients aged 65 years and older who were discharged alive after a hospitalization due to an osteoporotic hip fracture between 2008-2015. We used a Bayesian approach to approximate the posterior distribution of each parameter of the model, and thus cumulative incidences and transition probabilities. We also compared these results with a competing risks specification.

**Results:**

Posterior transition probabilities showed higher probabilities of death for men and increasing with age. Women were more likely to refracture as well as less likely to die after it. Free-event time was shown to reduce the probability of death. Estimations from the illness-death and the competing risks models were identical for those common transitions although the illness-death model provided additional information from the transition from refracture to death.

**Conclusions:**

We illustrated how multi-state models, in particular illness-death models, may be especially useful when dealing with survival scenarios which include multiple events, with competing diseases or when death is an unavoidable event to consider. Illness-death models via transition probabilities provide additional information of transitions from non-terminal health conditions to absorbing states such as death, what implies a deeper understanding of the real-world problem involved compared to competing risks models.

**Supplementary Information:**

The online version contains supplementary material available at 10.1186/s12874-023-01859-y.

## Background

Epidemiology deals with the incidence and evolution of diseases in populations. Although epidemiological studies can be conducted with different objectives and perspectives, time in these projects is an important, and often essential, element. Survival and event history analysis also address time and constitute important methodological tools for the treatment of epidemiological data. They focus on time to the occurrence of one or several events of interest which, in epidemiological research, are usually associated with death, the cure of a disease, a positive and/or negative progression of a disease, the appearance of adverse effects, etc. as well as with the possible trajectories determined by the occurrence of the different events in individuals of the target population.

Multi-state models are particular survival models developed for the joint statistical treatment of more than one event of interest that focus on the complete event history. They are a class of stochastic models that enable individuals to move between different states over time. These states generally represent different conditions of illness and/or health. Relevant survival times are times between states which, from a methodological perspective, can be analyzed by means of stochastic processes and survival procedures (See Andersen and Keiding [1]). Transition probabilities are the main outcome of those models. They can be used to assess progression of an individual between states based on the clinical history, which is especially useful for individualized health care. Despite their enormous usefulness, multi-state models are not yet very popular in the world of epidemiology [2–4]. Instead, competing risks models have been widely used to assess incidences and risks in the presence of competing events where the occurrence of a particular event prevents the occurrence of all remaining events, such as death [5–8].

Our aim in this paper is to propose a methodological Bayesian framework to connect the theoretical world of Bayesian multistate models with the world of biomedical research. We emphasise the potential of multi-state models to study epidemiological processes that require the analysis of different health conditions whose occurrence may be sequential in time and always in environments with uncertainty. Another important objective of this paper is to show the usefulness of Bayesian methods in the analysis of epidemiological data because they can express the dynamic information associated with key concepts relative to risks and rates in terms of probability distributions. In addition, we would show the connections between competing risks and multi-state models that allow us to clarify the usefulness of both models for analysing data from epidemiological settings as well as to deepen their analogies and differences.

This paper is structured as follows. First, we review competing risks and multi-state models, focusing on the *so-called* illness-death model, a particular type of multi-state model with three states, two of them transient and one terminal. Furthermore, we describe how transition probabilities are calculated in terms of the hazard functions from the survival framework. Next, we present a study based on data from the PREV2FO cohort, which comprises patients aged 65 years and older discharged after hospitalization for osteoporotic hip fracture between 2008 and 2015. We use an illness-death model to analyse the follow-up of these patients over time with special interest in the occurrence of a subsequent hip fracture and death. A Bayesian approach is performed to obtain posterior distributions of the cumulative incidences of refracture and death as well as of transition probabilities. The paper ends with some concluding remarks.

## Methods

### Competing risks models

Competing risks models are survival statistical models which consider many causes of failure [9]. Individuals in the study are followed from a common initial point until the occurrence of the first of the events considered. Such occurrence precludes the observation of the rest of events, acting as a censoring event.

**Fig. 1:**
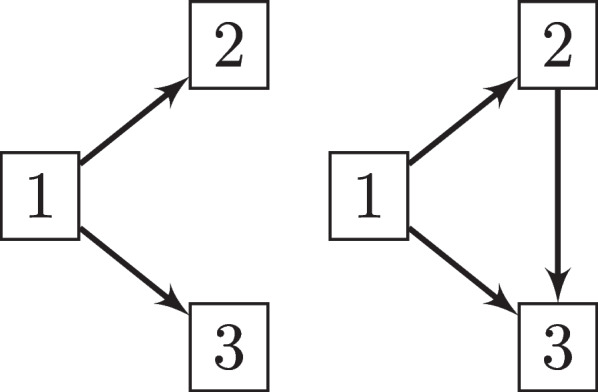
Diagram on the left: Competing risks model with two possible causes of failure (states 2 and 3). Diagram on the rigth: Illness-death model with an initial state (1), a transient state for illness (2), and an absorbing state such as death (3)

We assume, without loss of generality, a competing risk model with an initial common state, 1, and two possible events of interest, 2 and 3 (See Fig. 1). Let $T_ {12}$ and $T_{13}$ be the time from the starting state 1 to event 2 and 3, respectively and consider $T=\min \{T_{12}, T_{13}\}$ the time to the occurrence of the first event of the two competing events.

The two key concepts in competing risks models are the cause-specific hazard function, which assesses rates, and the cumulative incidence function for risks. Each can always be derived from the other.

The cause-specific hazard function of experiencing the event *j*, $j=2,3$ in the presence of the other competing event is defined as

1 $$\begin{aligned} h_{1j}(t)={\underset{\Delta t \rightarrow 0}{\lim}}\frac{P(t\leq T < t + \Delta t , \delta = j \mid T \geq t)}{\Delta t}, \quad t > 0, \end{aligned}$$

where $\delta = j$ is the value of the indicator for the occurrence of event *j*. Hazards functions are defined in terms of conditional probabilities but they are not proper probabilities because they can be greater than 1. In a completely intuitive and approximate way, we could interpret the hazard function of a given event at time *t* as the approximate instantaneous probability that an individual who has not experienced the event of interest at time instant *t* will experience it in a next small window of time.

Risks in competing risk models are mainly assessed by means of the cumulative incidence function. In the case of the event *j*, $j=2,3$ it is defined as the unconditional probability $F_{1j}(t)=P(T\leq t, \delta =j)$, which can be expressed in terms of hazard functions as

2 $$\begin{aligned} F_{1j}(t)=P(T\le t, \delta =j)=\int _0^t h_{1j}(u) \exp {\Big \{-\int _0^u \,\Big (h_{12}(s)+h_{13}(s)\Big ) \text{ d }s \Big \}} \,\text{ d }u \, . \end{aligned}$$

Cumulative incidence functions are very meaningful, as they can be interpreted as the proportion of individuals who failed by cause *j* before time *t*. They are the main outcomes of any competing risks analysis.

### Multi-state models

Multi-state models account for different structures depending on the number of states and how they relate to each other [1]. We focus on a particular type of multi-state model known as the illness-death or disability model. This is a multi-state model with three states, which generally represent different health conditions, the starting state 1 is usually health, the event 2 is an intermediate state generally associated to illness, and state 3 is terminal and it is generically associated to death. A healthy person can go directly to suffering from a certain illness or die without having suffered from that illness. But it is also possible that they die after having suffered from the illness. We are now faced with two competitive events but one of them is of a transitory nature and allows access to the terminal event death (See Fig. 1). This model makes possible to study the risk of death whether the individual has been previously ill or not.

The methodological framework of multi-state models are the stochastic processes in continuous time and finite state space, the latter defined by the events of interest of the study. Its stochastic treatment is based on the concepts and procedures of this field. In this regard, the so called transition probabilities between the states determine the probabilistic behavior of the illness-death model. They connect the behavior of the process in two different times in terms of conditional probabilities as follows,

3 $$\begin{aligned} p_{ij}(s,t)=P(X(t)=j \mid X(s)=i), \end{aligned}$$

defined as the probability of being at the state *j* at time *t* $(X(t)=j)$ given that the same individual was in state *i* at *s* $(X(s)=i)$.

The world of stochastic processes and the world of survival analysis are connected: transition probabilities between states determine the hazard functions of the relevant survival time in full and vice versa. In this regard, if we move to an illness-death model within a survival scenario we could focus on survival times from state 1 to 2, from 1 to 3, and from 2 to 3 and their subsequent hazard functions. The specific linkage between transition probabilities and hazard functions is as follows (Armero et al. [10]):

4 $$\begin{aligned} p_{11}(s,t)= & {} \exp {\Big \{-\int _s^t \,\Big (h_{12}(u)+h_{13}(u)\Big ) \text{ d }u \Big \}}\nonumber \\ p_{22}(s,t \mid t_{12})= & {} \exp {\Big \{-\int _s^t h_{23}(u-t_{12}|t_{12})\text{ d }u \Big \}} \nonumber \\ p_{12}(s,t)= & {} \int _s^t p_{11}(s,u) h_{12}(u)p_{22}(u,t| u) \text{ d }u \nonumber \\ p_{13}(s,t)= & {} 1-p_{11}(s,t)-p_{12}(s,t)\nonumber \\ p_{23}(s,t \mid t_{12})= & {} 1-p_{22}(s,t \mid t_{12})\nonumber \\ p_{33}(s,t)= & {} 1, \end{aligned}$$

Some relevant transition probabilities are $p_{11}(0,t)$, which assesses the permanence of the process in the initial state at time *t* and therefore the lack of occurrence of any event in that period, $p_{12}(0,t)$ and $p_{13}(0,t)$, which provide the probability of being in state 2 and 3 at time *t*, respectively, and connect with the cumulative incidence function for event 2 and 3, respectively, and $p_{22}(s,t\mid t_{12})$ which quantifies the permanence in the illness state at time *t* given that illness has occurred previously at a known time $t_{12}$. Those transition probabilities are especially useful for an individualized health care. As they consider information on the clinical history of a particular patient, it is possible to make predictions, via those probabilities, on the future health condition of a patient.

All of those probabilities are defined in a time-since-fracture scale, being $t_{12}$, *s*, and *t* times from fracture. Transition probability $p_{23}(s,t\mid t_{12})$ can, however, be rescaled to time-since-refracture by fixing $s=t_{12}$. As a result, this probability only depends on $t-t_{12}$, which is indeed the transition time from 2 to 3, being it possible to focus only on this difference. Moreover, other starting points can be set, such as $s=t_{12}+1$ or $s=t_{12}+2$, thus assessing the probability of dead for those who survived for 1 or 2 years after entering illness state, respectively.

Competing risk models can be considered as multi-state models with two types of events: an initial state and two states for the competing causes of failure which are terminal and without connection among them [11]. Relevant transitions between states are the ones that start from the initial state to each of the other two states.

### Bayesian inference

Bayesian statistics is considered to approach multi-state models and transition probabilities. Bayesian inference is a statistical methodology based on a conception of probability that allows to express the uncertainty associated to any element of a statistical model such as the unknown parameters in terms of probability distributions. Bayesian inference combines the prior knowledge of those parameters, $\boldsymbol{\theta }$, and the experimental data. The Bayesian inferential process is thus sequential: first, a prior distribution of the parameters, $\pi (\boldsymbol{\theta })$, is elicited; information from data $\mathcal D$ is added through the derived likelihood function; and, finally, the knowledge of the parameters is updated via Bayes theorem and included in the posterior distribution, $\pi (\boldsymbol{\theta }\mid \mathcal {D})$. The choice of a prior distribution depends on the available information of parameters and has been object of extensive discussion [12–14]. In particular, non-informative priors are used when no prior information is available or the main interest is to assess information from data alone.

The posterior distribution $\pi (\boldsymbol{\theta }\mid \mathcal {D})$, which contains all the relevant information about the parameters of the model, is usually not analytical and can be approximated by numerical methods. In this sense, Markov Chain Monte Carlo methods (MCMC) and the integrated nested Laplace approximation (INLA) are the more popular computational methods for this issue. The former are simulation procedures that approximate the joint posterior distribution by mean of a claver Markov chain that converges to the target posterior distribution [15]. The latter method computes approximations of the posterior marginal distributions for the parameters and hyperparameters in *latent Gaussian models*, a subset of models which includes generalized regression models, smoothing splines models, spatial and survival models, among others [16].

On the other hand, the posterior distribution for any output of interest that is a function of the model parameters can be approximated by the simulated sample of the posterior $\pi (\boldsymbol{\theta }\mid \mathcal {D})$. For instance, in the illness-death model, the posterior distribution of cumulative incidences, $\pi (F_{1j}(t)\mid \mathcal {D})$ , and transition probabilities, $\pi (p_{ij}(s,t)\mid \mathcal {D})$, can be approximated. Note that those distributions contain full information of those quantities in probabilistic terms and thus posterior mean, median, quantiles and credible intervals can be calculated.

### Refracture and death after an osteoporotic hip fracture: the PREV2FO study

Hip fracture is a main complication of osteoporosis in elder populations. Beyond its economic cost for health care systems and society, it results in significant reductions in quality of life, disability, morbidity and mortality [17–19]. Patients after a hip fracture are at much higher risk of a subsequent hip fracture [20, 21] as well as death, and excess of mortality is even higher after a second hip fracture [22, 23].

We considered the PREV2FO cohort, a population-based cohort of patients aged 65 years and older, discharged alive after hospitalization for osteoporotic hip fracture from January 1, 2008, to December 31, 2015. Patients were followed from the date of discharge (time zero) to December 31, 2016 (end of study) or death. The study was conducted in the region of Valencia in Spain (around 5 million inhabitants, representing 10% of the whole country population) providing free, universal health care services (besides drug cost-sharing) to 97% of the region’s population. Data were obtained from the Valencia Health System Integrated Database (VID). VID is the result of the linkage, by means of a single personal identification number, of a set of publicly owned population-wide health care, clinical, and administrative electronic databases in Valencia, which has provided comprehensive information since 2008 [24].

### A Bayesian illness-death model for refracture and death after hip fracture

**Fig. 2:**
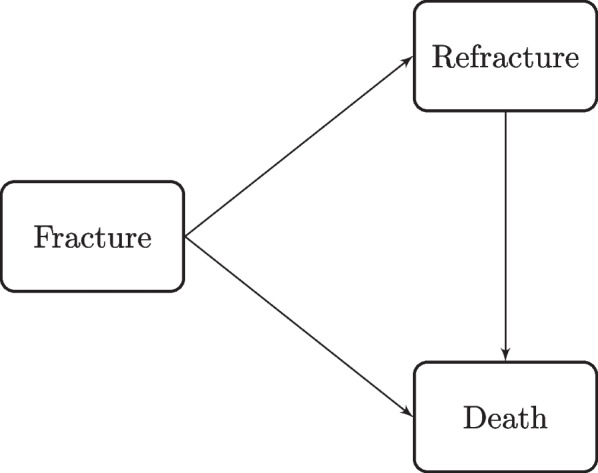
Illness-death model with an initial state of hip fracture, a recurrent hip fracture state and a death state

We model the possible trajectories of the patients in the PREV2FO study by means of an illness-death model. The initial status of all patients in the study is a hip fracture from which they have recovered in hospital. The two remaining events of interest are a new hip fracture, which we will call a refracture state, or death. The estimation of the incidence of recurrent hip fractures and death after an osteoporotic hip fracture, one of the main objectives of the study, could be approached by means of a competing risk model if the refracture condition were considered as a terminal event which competes with the risk of death. This is not the case because the refracture condition is considered in the study as a transitory state towards death (See Fig. 2).

Relevant survival times in this model are the time $T_{FD}$ from discharge after fracture (*F*) to death (*D*), the time $T_{FR}$ from discharge after fracture to refracture (*R*), and the time from refracture to death, $T_{RD}$.

Cox proportional hazards models [25] with Weibull baseline hazard functions, and gender and age at discharge as covariates have been used to model the random behavior of the relevant times, $T_{FR}$, $T_{FD}$, and $T_{RD}$, in the study. In addition, transition from refracture to death requires to be modeled with consideration to the time when patients refractured, defining a semi-Markov model for this transition.

The modeling for each transition includes four unknown parameters. Two parameters are the shape and scale of the baseline Weibull distributions $\alpha ^{(ij)}$ and $\lambda ^{(ij)}$, respectively, for each transition from *i* to *j*. On the other hand, a total of two parameters were considered as regression coefficients for covariates sex and age, $\beta _{Wo}^{(ij)}$ because the reference group included in the baseline hazard corresponds to men, and $\beta _{Age}^{(ij)}$, respectively. Given those parameters the illness-death model can be defined in terms of the hazard functions as:

5 $$\begin{aligned} h^{F\!R}(t)= & {} h^{F\!R}_0(t)\exp \{\beta ^{F\!R}_{Wo} \text {I}_{Wo}+\beta ^{F\!R}_{\text {Age}} \text {Age}\},\nonumber \\ h^{F\!D}(t)= & {} h^{F\!D}_0(t)\exp \{\beta ^{F\!D}_{Wo} \text {I}_{Wo}+\beta ^{F\!D}_{\text {Age}} \text {Age}\},\nonumber \\ h^{R\!D}(t-t_{F\!R}\mid T_{F\!R} = t_{F\!R})= & {} h^{R\!D}_0(t-t_{F\!R}\mid T_{F\!R}=t_{F\!R})\exp \{\beta ^{R\!D}_{Wo} \text {I}_{Wo}+\beta ^{R\!D}_{\text {Age}} \text {Age}\}, \end{aligned}$$

where $\text {I}_{Wo}$ is the indicator variable which takes the value 1 if the patient is a woman and 0 otherwise, and $h_{0}$ are the baseline hazard functions in defined as

6 $$\begin{aligned} h^{F\!R}_0(t)= & {} \alpha ^{(FR)}\lambda ^{(FR)} t^{\alpha ^{(FR)}-1},\nonumber \\ h^{F\!D}_0(t)= & {} \alpha ^{(FD)}\lambda ^{(FD)} t^{\alpha ^{(FD)}-1},\nonumber \\ h^{R\!D}_0(t-t_{FR}\mid T_{FR}=t_{FR})= & {} \alpha ^{(RD)}\lambda ^{(RD)} (t-t_{FR})^{\alpha ^{(RD)}-1}. \end{aligned}$$

We used a Bayesian approach to estimate the parameters of the illness-death model. Consequently, we have followed the usual steps of the Bayesian inferential process: specification of a prior distribution for the parameters of the model, construction of the likelihood function, and computation of the posterior distribution via Bayes’ theorem.

The prior distribution is elicited assuming a prior independence and non-informative scenario. Wide normal prior distributions were selected for the regression coefficients and Gamma distributions for the shape and scale parameters of the Weibull baseline hazards (See Alvares et al. [26] for a wide tutorial on Bayesian survival models). Posterior distributions were approximated via MCMC methods as well as through the INLA. In particular, age as a covariate in our model is included as mean-centered in order to improve the convergence of the subsequent computational MCMC methods. Computation has been done using JAGS [27] and R [28].

As complementary analyses, frequentist non-parametric estimators were calculated to compare their results with those obtained from the Bayesian illness-death model with Weibull hazards. Nelson-Aalen estimator [29, 30] provided similar numerical results to the estimated cumulative hazards from the Weibull model. On the other hand, similarities between cumulative incidences from the parametric model and the Aalen-Johansen estimator, which is a multi-state version of Kaplan-Meier estimator [31], suggest a good fit of the proposed model with Weibull distributions (See Additional file 1).

## Results

### Hip fracture data description

The study involved 34,491 patients discharged alive after hip fracture, 25,807 (74.8%) were women and 8684 (25.2%) men. Regarding age, 12.4% of patients were under 75 years old, 43.6% between 75 and 85 years old, 40.6% between 85 and 94 years old, and 3.4% were over 95 years old. The mean age at the first fracture was 83.4 years (IQR: 79.0-88.3). Patients were followed a median time of 5.0 years (IQR: 3.0-7.0 years).

### Posterior estimates

The approximate posterior distribution for the parameters of the illness-death model has been summarized in Table 1 via their posterior mean and standard deviation. We have also analyzed the data by means of a competing risk model. No differences have been observed between the results of the competing risk and the illness-death model for the two common transitions, from fracture to refracture and from fracture to death. However, as discussed above, the illness-death model provides additional information regarding the transition from refracture to death. The posterior mean associated with age is positive for all three transitions indicating a positive increase in the hazard corresponding to each event with age, although the most relevant relationship is for the time from fracture to death. Being a woman increases, with respect to men, the risk of fracture but decreases the risk of death, both from the initial fracture and after having experienced a refracture.

**Table 1:** Summary of the approximate posterior distribution of the parameters for the illness-death model

Time	Parameter	Mean	SD
From *F* to *R*	$\alpha ^{(F\!R)}$	0.9198	0.0157
	$\lambda ^{(F\!R)}$	0.0279	0.0013
	$\beta _{Wo}^{(F\!R)}$	0.0262	0.0486
	$\beta _{Age}^{(F\!R)}$	0.0244	0.0030
From *F* to *D*	$\alpha ^{(F\!D)}$	0.7759	0.0051
	$\lambda ^{(F\!D)}$	0.3311	0.0050
	$\beta _{Wo}^{(F\!D)}$	-0.5092	0.0166
	$\beta _{Age}^{(F\!D)}$	0.0705	0.0012
From *R* to *D*	$\alpha ^{(R\!D)}$	0.6234	0.0154
	$\lambda ^{(R\!D)}$	0.5769	0.0329
	$\beta _{Wo}^{(R\!D)}$	-0.6127	0.0655
	$\beta _{Age}^{(R\!D)}$	0.0498	0.0046

### Cumulative incidences

The cumulative incidence function for refracture (death) assesses the probability that a person refractures (dies) before a certain time. Both probabilities depend on the parameters of the model and consequently their posterior distribution is determined by the posterior distribution computed previously. Competing risk and illness-death models provide almost identical results for the incidence of refracture and death without refracture (See Table 2, posterior mean of the one-year cumulative incidence of refracture and death without refracture by sex and age). As we know, the illness-death model provides additional information regarding the probability of transition from refracture to death (the transition probability of death after refracture).

**Table 2:** One-year cumulative incidence of refracture, death without refracture and death after refracture. Table contains the posterior mean and a 0.95 credibility interval in percentage scale, by sex and age. Cumulative incidence of death after refracture equals to the transition probability of death after refracture at $s=0$

			Illness-death model	
Time	Sex	Age	Mean	95% CI
From *F* to *R*	Women	70	1.96	(1.76, 2.16)
		80	2.39	(2.24, 2.55)
		90	2.80	(2.60, 3.00)
	Men	70	1.86	(1.65, 2.09)
		80	2.21	(2.01, 2.42)
		90	2.45	(2.21, 2.72)
From *F* to *D*	Women	70	7.36	(7.05, 7.67)
		80	14.30	(13.95, 14.63)
		90	26.72	(26.17, 27.26)
	Men	70	11.95	(11.43, 12.46)
		80	22.63	(22.02, 23.29)
		90	40.35	(39.42, 41.36)
From *R* to *D*	Women	70	14.81	(12.81, 16.95)
		80	23.15	(21.51, 24.83)
		90	35.16	(32.79, 37.54)
	Men	70	25.50	(22.02, 29.43)
		80	38.49	(35.02, 42.07)
		90	55.04	(50.55, 59.51)

Overall, refracture probability increases with age and women have also higher incidence (one-year incidences of 1.96% at 70 and 2.80% at 90 years old for women, whilst 1.86% and 2.45% for men). Regarding death without refracture, incidence increases with age and has been estimated higher for men (one-year incidences of 7.36% at 70 and 26.73% at 90 years old among women, *versus* 11.94% and 40.34% for men).

### Transition probabilities

**Fig. 3:**
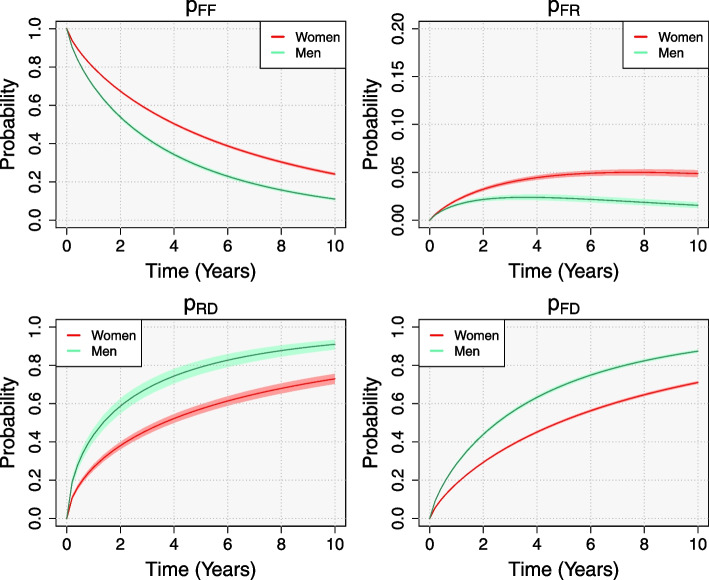
Posterior mean and 0.95 credibility intervals of some relevant transition probabilities (from top-left to bottom-right): event-free probability ($p_{F\!F}$) counting from discharge after the first hip fracture; transition probability from the initial fracture state to refracture state ($p_{F\!R}$) counting from first fracture; probability of death after refracture ($p_{R\!D}$) counting from discharge after refracture; and total probability of death ($p_{F\!D}$) counting from first fracture. Up to 10 years, by sex, for patients aged 80 years old. Time scale is time since fracture for $p_{F\!F}$, $p_{F\!R}$, $p_{F\!D}$, and time since refracture for $p_{R\!D}$

The first of the estimated transition probabilities in Fig. 3 is the event-free probability, or the transition probability of remaining at the initial state (fracture) without any progression, either refracture or death. Women show less events than men; mean event-free probabilities after 5 years were estimated at 51.69% and 36.12%, respectively, for patients aged 80 years. In addition, the older patients are, the more likely they are to progress, as both the risks of death and refracture increase with age.

**Fig. 4:**
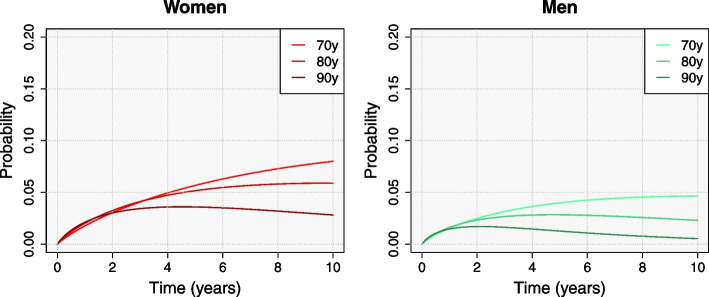
Posterior mean of the transition probability from the initial fracture state to refracture state ($p_{F\!R}$) up to 10 years after discharge, by sex and age

In the second place, we estimated the transition probability from fracture to refracture ($p_{F\!R}$ from Fig. 3). Women showed higher mean transition probabilities than men. For patients aged 80 years at discharge, we expect 2.02% of women to have a second fracture and be still alive 1 year after the first fracture, compared with a 1.62% of men (5.16% and 2.84% after 5 years, respectively). This derives from higher mortality rates among men: a higher competing risk of death results in less refractures, whereas higher probabilities of death after refracture decrease men’s permanence at the refracture state. For the same reason, as the age increases, the transition probability decreases (Fig. 4). The expected time-trends also differ among age groups. As mortality among patients aged 70 years is fairly low compared to older populations, we expect the proportion of patients at the refracture state to increase over time. On the contrary, the proportion of patients aged 90 years with a refracture and still alive decreases after the 2nd year for men and after the 5th for women.

Finally, we estimated the probability of death after refracture, $p_{R\!D}$, and the total probability of death, $p_{F\!D}$, which includes deaths from both pathways, without and after refracture. In broad terms, the transition probability of death after refracture shows a similar pattern to death without refracture, i.e increases with age and is higher for men: one-year probabilities of 14.81% at 70 and 35.16% at 90 years old for women, whereas 25.50% and 55.04% for men. Regarding the total probability of death, higher mortality probabilities were estimated for men than for women, increasing with age. One year after the initial fracture the probability of death was estimated at 7.55% and 27.39 % for women at 70 and 90 years old, respectively, whilst 12.27% and 41.34% for men. Those total probabilities of death are only estimable after considering the transition from refracture to death, as the illness-death model does.

### Conditioned probabilities

It is important to note that each transition probability until now has been calculated starting from the event occurrence $(s=0)$. We estimated the probability of death one year after the refracture, and the total probability of death one year after the initial fracture. However, the potential of the transition probabilities is much greater as their starting time can be defined at any time during the study period and thus not only starting at the event occurrence. In particular, we can estimate those transition probabilities after being a certain amount of time without event. For instance, we can consider an event-free situation one year after $(s=1)$ the start of the process, and thus estimate the probability of death after refracture at *t*, considering that no event was observed the year after the refracture, and the total probability of death at *t* given no event the year after the initial fracture. It can be seen that surviving a year after discharge (for both fracture and refracture) decreases mortality.

One-year transition probabilities of death after refracture for those patients who were event-free 1 year after refracture (i.e. after a total of 2 years from discharge after the recurrent fracture) were estimated at 8.30% at 70 and 20.88% at 90 years old for women, whereas 14.78% at 70 and 35.10% at 90 years old for men.

On the other hand, we estimated one-year total probabilities of death for those patients who were event-free 1 year after the first fracture (i.e. after a total of 2 years after the first fracture) of 5.47% at 70 and 20.50% at 90 years old for women, whilst 8.95% at 70 and 31.77% at 90 years old for men.

**Fig. 5:**
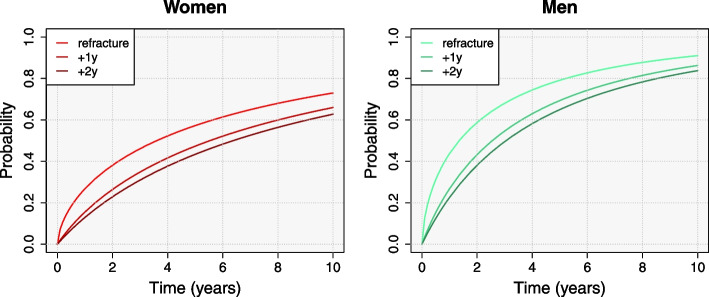
Posterior mean of the probability of death after refracture ($p_{R\!D}$) up to 10 years counting from different starting time points: discharge after refracture, assuming that patients survived at least 1 year after refracture, and assuming that they survived at least 2 years after refracture. By sex, for patients aged 80 years old

For illustrative purposes, we also depict the variation in the probability of death after refracture for those patients who survive 1 year and 2 years since being discharged after refracture (Fig. 5). It shows how the more time patients survive after refracture the less likely they are to die. Moreover, the differences in those probabilities of death decrease when increasing the known time patients stay alive.

### Competing risks vs illness-death

**Fig. 6:**
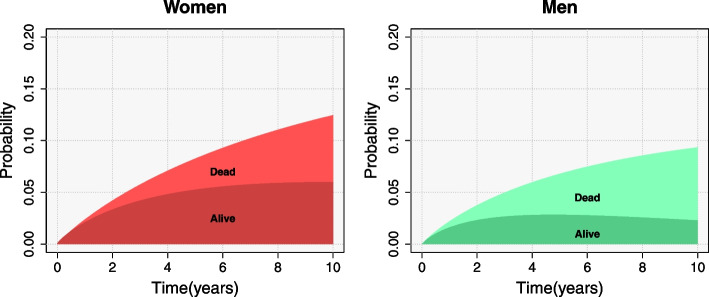
Posterior mean of the cumulative incidence of refracture (upper curve) and of the transition probability from initial state to refracture state (lower curve), for patients aged 80 years old, by sex. Light area in both figures represents the proportion of dead patients who had previously suffered a refracture while the dark area represents the proportion of refractured people still alive

Our illness-death model provides relevant information about the transition from refracture to death that the competing risks model cannot address. Figure 6 shows the posterior mean of the cumulative incidence of refracture and the posterior mean of the transition probability from the initial state of fracture to the refracture state. Both outcomes are defined in relation to a specific time *t*. The upper curve corresponds to the total accumulation of refractures (cumulative incidence) occurred before time *t* and the lower curve refers to the probability that a patient is in the state of refracture at time *t*, and therefore alive. Thus, it should be understood that at time *t* the dark shaded area shows living patients who have refractured and the light shaded area shows refractured patients who have died.

## Discussion

We illustrated how multi-state models, in particular illness-death models, may be especially useful when dealing with survival scenarios which include multiple events, such as competing diseases, or when death is an unavoidable event to consider. Illness-death models provide not only the same information as competing risks models but also information of the transition from illness to death.

Transition probabilities are obtained from multi-state models as natural, rich and dynamic outcomes. They provide clear evidence about progression and the trajectories that patients follow from the moment they enter the study. On the other hand, probabilities can be estimated based on the clinical history of a particular individual. As a result, transition probabilities may be especially useful for an individualized health care, allowing to make sequential and updated predictions on their future health conditions during the follow-up.

Regarding the PREV2FO study, it is worth mentioning that narrow credible intervals were estimated for transition probabilities. Probabilities which include only refracture and death after refracture, $p_{FR}$ and $p_{RD}$, showed more variability and wider intervals. A possible reason would be the large sample size, 34 491 patients, which would allow the model to be quite confident in its posterior inference. It seems particularly true for death without refracture due to the high mortality rates and less censoring. Few patients refracture, 2 532 (7.3% of the total), which reduces the number of patients at risk of death after refracture. Thus, it would increase the uncertainty and lead to wider credible intervals.

An important consideration should be made about the estimation of the incidence of death among the population. Although the incidence of refracture can be estimated equally from both illness-death and competing risks models, that is not the case with the incidence of death. The proposed competing risks model only accounts for death without refracture, as the observation of death is censored for those refractured patients. However, when including death after refracture through the illness-death model, the total incidence of death becomes a natural outcome. In particular, it is defined as the transition probability from the initial state to death state.

Note that we provided estimations of some selected transition probabilities in order to illustrate their potential. Nevertheless, we could estimate an endless number of transition probabilities by means of modifying the desired time intervals and conditioning on different event-free times. It allows to formulate and answer a wide range of scientific questions, providing a better understanding of real-world problems. For example, the flexibility of those multi-state models would allow to include another state for a second refracture, or reversible transitions, in case they made sense.

The multi-state methodology included in our work is easily generalizable. We used a full parametric approach via Weibull hazard functions. However, semi-parametric specifications of baseline hazards can be a good choice for Bayesian Cox models [32, 33]. On the other hand, residual testing from a Bayesian perspective is complicated [34]. However, the basic model can be modified to better reflect the real trend in the data. Stratified Cox proportional hazards models would be an option in case that proportionality could not be assumed for some covariates [35]. Non-linear effects of predictors can also be modelled, including regression splines [36], for instance.

Homogeneity have been tested by including the time from fracture to refracture in the model as a covariate. The associated coefficient was estimated non-relevant, which suggests that the homogeneous model would be a reasonable choice.

Finally, it is important to mention that the Bayesian approach eases the whole inferential process, making it possible to estimate posterior distributions of each function of the parameters of the model, such as hazard ratios, cumulative incidences, and transition probabilities. It makes the statistical inference more natural and intuitive, given this strongly probabilistic approach.

All of these advantages, always under the aforementioned assumptions and considerations, make the illness-death model, in particular from a Bayesian perspective, a preferable tool/method to assess scenarios which include more than one event or when death is an unavoidable event to consider (e.g. elder population).

## Conclusions

Multi-state models, in particular illness-death models, may be especially useful when dealing with survival scenarios which include multiple events, such as competing diseases, or when death is an unavoidable event to consider, with transition probabilities as rich and dynamic outcomes. A Bayesian approach is a good choice to deal with multi-state models due to its interpretability, and provides a flexible framework which can be easily generalized.

## Supplementary Information

**Additional file 1.** Comparing Nelson-Aalen and Aalen-Johansen estimators versus estimates from a Bayesian approach to the illness-death model with Weibull times.

**Additional file 2.** R code. JAGS model and estimation of cumulative incidences and transition probabilities.

## Data Availability

The data that support the findings of this study are not publicly available due to legal restrictions on sharing the data set, regulated by the Valencia regional government by means of legal resolution by the Valencia Health Agency [2009/13312]. Data are however available from the authors upon reasonable request. Requests to access the datasets should be directed to Management Office of the Data Commission in the Valencia Health Agency (email: solicitud_datos@gva.es). R code with the JAGS model and functions for estimating cumulative incidences and transition probabilities has been included in Additional file 2.
