# Supplementary material for: Estimating disease incidence rates and transition probabilities in elderly patients using multi-state models: a case study in fragility fracture using a Bayesian approach
Source: BMC Med Res Methodol. 2023 Feb 14;23:40. doi: 10.1186/s12874-023-01859-y (PMC9930279; doi:10.1186/s12874-023-01859-y)
Supplement: Supplementary file 1 — Additional file 1. Comparing Nelson-Aalen and Aalen-Johansen estimators versus estimates from a Bayesian approach to the illness-death model with Weibull times. [file 12874_2023_1859_MOESM1_ESM.pdf]

Additional file 1: Comparing Nelson-Aalen and Aalen-Johansen estimators versus estimates from a Bayesian approach to the illness-death model with Weibull times.

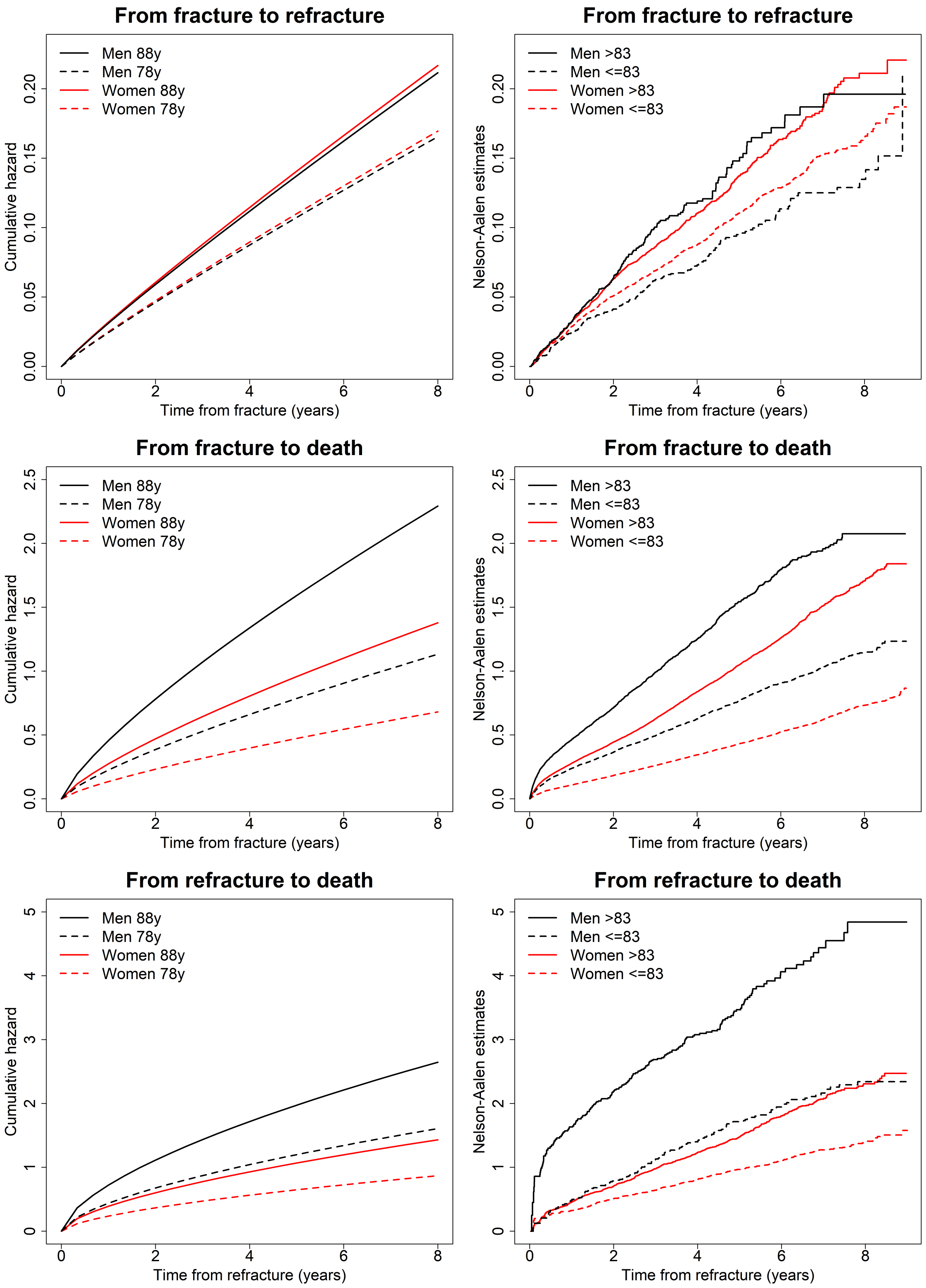

Additional figure 1: Cumulative hazards from a Bayesian illness-death model versus Nelson-Aalen estimator. (Left column) Posterior mean of the cumulative hazards from a Bayesian Weibull illness-death model, associated to transitions from fracture to refracture, from fracture to death, and from refracture to death. Predicted curves by sex, and for individuals aged 78 and 88 years. (Right column) Nelson-Aalen estimator associated to the same transitions. Grouped by sex and age (≤ 83 or > 83 years old).

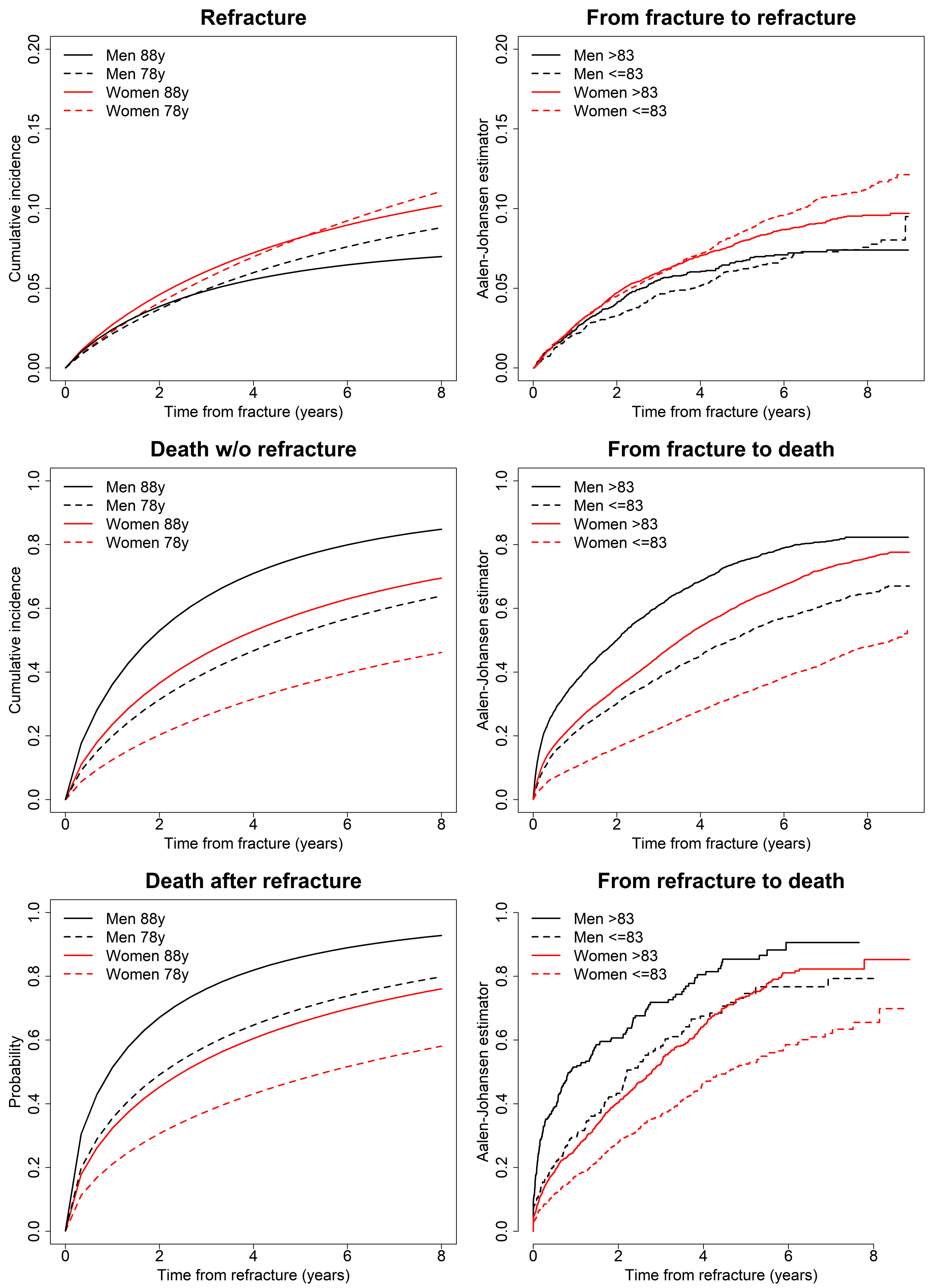

Additional figure 2: Cumulative incidences from a Bayesian illness-death model versus Aalen-Johansen estimator. (Left column) Posterior mean of the cumulative incidences of refracture and death without refracture, and transition probability of death after refracture, from a Bayesian Weibull illness-death model. Predicted curves by sex, and for individuals aged 78 and 88 years. (Right column) Aalen-Johansen estimator associated to the same transitions. Grouped by sex and age (≤ 83 or > 83 years old).
