# Supplementary material for: Estimating disease incidence rates and transition probabilities in elderly patients using multi-state models: a case study in fragility fracture using a Bayesian approach
Source: BMC Med Res Methodol. 2023 Feb 14;23:40. doi: 10.1186/s12874-023-01859-y (PMC9930279; doi:10.1186/s12874-023-01859-y)
Supplement: Supplementary file 2 — Additional file 2. R code. JAGS model and estimation of cumulative incidences and transition probabilities. [file 12874_2023_1859_MOESM2_ESM.pdf]

Additional file 2: R code. JAGS model and estimation of cumulative incidences and transition probabilities.

1 Multi-state model JAGS

```
#MULTI-STATE MODEL JAGS

weib.ms.model<- "model{

#Total likelihood

  for(k in 1:n){

    #Hazard and log-survival functions
     #Transition 1,2
      base[1,2,k] <- lambda[1,2]*alpha[1,2]*pow(t[k,1],alpha[1,2]-1)
      elinpred[1,2,k] <- exp(inprod(beta[,1,2], X[k,]))
      h[1,2,k] <- base[1,2,k]*elinpred[1,2,k]
      logsurv[1,2,k]<- -lambda[1,2]*pow(t[k,1], alpha[1,2])*elinpred[1,2,k]

     #Transition 1,3
      base[1,3,k] <- lambda[1,3]*alpha[1,3]*pow(t[k,1],alpha[1,3]-1)
      elinpred[1,3,k] <- exp(inprod(beta[,1,3], X[k,]))
      h[1,3,k] <- base[1,3,k]*elinpred[1,3,k]
      logsurv[1,3,k]<- -lambda[1,3]*pow(t[k,1], alpha[1,3])*elinpred[1,3,k]


     #Definition of the log-likelihood using zeros trick
      trans12[k]<- c[k,1]*log(h[1,2,k])+logsurv[1,2,k]
      trans13[k]<- c[k,2]*log(h[1,3,k])+logsurv[1,3,k]
```

```
      phi[k] <- 100000-(trans12[k]+trans13[k]+trans23[k])
      zeros[k] ~dpois(phi[k])
  }

  #Likelihood for transition 2,3

  for(j in fracts){

    base[2,3,j] <- lambda[2,3]*alpha[2,3]*pow(t[j,2]-t[j,1],alpha[2,3]-1)
    elinpred[2,3,j] <- exp(inprod(beta[,2,3], X[j,]))
    h[2,3,j] <- base[2,3,j]*elinpred[2,3,j]
    logsurv[2,3,j]<- -lambda[2,3]*pow(t[j,2]-t[j,1], alpha[2,3])*elinpred[2,3,j]

    trans23[j] <- c[j,3]*log(h[2,3,j])+logsurv[2,3,j]
  }

  for(p in nofracts){

      trans23[p]<- 0
  }

  #Prior distributions
    for(l in 1:Nbetas){
    beta[l,1,2] ~dnorm(0,0.01)
    beta[l,1,3] ~dnorm(0,0.01)
    beta[l,2,3] ~dnorm(0,0.01)
    }

    lambda[1,2] ~ dgamma(0.01,0.01)
    lambda[1,3] ~ dgamma(0.01,0.01)
    lambda[2,3] ~ dgamma(0.01,0.01)

    alpha[1,2] ~ dgamma(0.01,0.01)
    alpha[1,3] ~ dgamma(0.01,0.01)
    alpha[2,3] ~ dgamma(0.01,0.01)

}"
```

```
#MCMC SAMPLES (Paralleled)
#Data
d.jags <- list(n=nrow(X), t=time, X=X, c=c, zeros=rep(0,nrow(X)), Nbetas=ncol(X),
               fracts=which(c[,1]==1), nofracts=which(c[,1]==0))
#Parameters
p.jags <- c("beta", "alpha", "lambda")

#Wrapper for parallelization
coda.samples.wrapper <- function(j)
{
  m1 = jags.model(data=d.jags, file=textConnection(weib.ms.model),
                  inits=list(.RNG.name="base::Wichmann-Hill", .RNG.seed=j),
                  n.chains=1)
  update(m1, 1000)
  coda.samples(m1, variable.names=p.jags, n.iter=10000, thin=1)
}
```

2 Cumulative incidences and transition probabilities

2.1 Cumulative incidence of refracture

```
#General notation. States: 1 fracture, 2 refracture, 3 death. Transitions:
#"12" transition from fracture to refracture, "13" transition from fracture
#to death, "23" transition from refracture to death.
#Regression coefficients: beta1 (sex) and beta2 (age) for each transition,
#e.g. beta112 effect of sex on risk of refracture (transition 12).


cif1 <- function(t,x){

  media<- numeric(length(t))
  quantiles<- matrix(nrow=2,ncol=length(t))

  x[2]<- x[2]-mean(edad_i)

  exp12<- exp(x[1]*beta112+x[2]*beta212) #regression terms
```

```
  exp13<- exp(x[1]*beta113+x[2]*beta213)

  int<- numeric(length(alpha12))   #integral
  ext<- lambda12*alpha12*exp12     #exterior part

  for(i in 1:length(t)){ #for each given time

    for(j in 1:length(alpha12)){
    #for each sample from MCMC -> whole posterior distribution

      ff<-function(u){u^(alpha12[j]-1)*exp(-lambda12[j]*u^alpha12[j]*exp12[j]-
                                             lambda13[j]*u^alpha13[j]*exp13[j])}
      int[j]<-integrate(ff,0,t[i],rel.tol = 1e-8)[[1]]
    }

    media[i]<- mean(ext*int)
    quantiles[,i] <- quantile(ext*int, probs=c(0.025,0.975))

  }

  list(media,quantiles)
}
```

2.2 Cumulative incidence of death without refracture

```
cif2 <- function(t,x){

  media<- numeric(length(t))
  quantiles<- matrix(nrow=2,ncol=length(t))

  x[2]<- x[2]-mean(edad_i)

  exp12<- exp(x[1]*beta112+x[2]*beta212) #regression terms
  exp13<- exp(x[1]*beta113+x[2]*beta213)

  int<- numeric(length(alpha13)) #integral
  ext<- lambda13*alpha13*exp13   #exterior part
```

```
  for(i in 1:length(t)){ #for each given time

    for(j in 1:length(alpha12)){
    #for each sample from MCMC -> whole posterior distribution

      ff<-function(u){u^(alpha13[j]-1)*exp(-lambda12[j]*u^alpha12[j]*exp12[j]-
                                         lambda13[j]*u^alpha13[j]*exp13[j])}
      int[j]<-integrate(ff,0,t[i],rel.tol = 1e-8)[[1]]
    }

    media[i]<- mean(ext*int)
    quantiles[,i] <- quantile(ext*int, probs=c(0.025,0.975))

  }

  list(media,quantiles)
}
```

2.3 Event-free probability, $p_{FF}(s,t)$

```
p11.func <- function(s,t,x){
  media<-numeric(length(t))
  quantiles<- matrix(nrow=2,ncol=length(t))

  x[2]<- x[2]-mean(edad_i)

  for(i in 1:length(t)){
    aux<- exp(-lambda12*(t[i]^alpha12-s^alpha12)*exp(x[1]*beta112+x[2]*beta212)-
              lambda13*(t[i]^alpha13-s^alpha13)*exp(x[1]*beta113+x[2]*beta213))

    media[i]<-mean(aux)
    quantiles[,i] <- quantile(aux, probs=c(0.025,0.975))
  }

  list(media, quantiles)

}
```

2.4 Transition probability from fracture to refracture, $p_{FR}(s,t)$

```
p12.func <- function(s,t,x){

  media<- numeric(length(t))
  quantiles<- matrix(nrow=2,ncol=length(t))

  x[2]<- x[2]-mean(edad_i)

  exp12<-exp(x[1]*beta112+x[2]*beta212)
  exp13<-exp(x[1]*beta113+x[2]*beta213)
  exp23<-exp(x[1]*beta123+x[2]*beta223)

  int<-   numeric(length(alpha12))
  ext<- lambda12*alpha12*exp12*exp(lambda12*s^alpha12*exp12+
                                   lambda13*s^alpha13*exp13)

  for(i in 1:length(t)){

    for(j in 1:length(alpha12)){

    ff<-function(u){
            u^(alpha12[j]-1)*exp(-lambda12[j]*u^alpha12[j]*exp12[j]-
                                  lambda13[j]*u^alpha13[j]*exp13[j]-
                                  lambda23[j]*(t[i]-u)^alpha23[j]*exp23[j])}
    int[j]<-integrate(ff,s,t[i],rel.tol = 1e-8)[[1]]
    }

    media[i]<- mean(ext*int)
    quantiles[,i] <- quantile(ext*int, probs=c(0.025,0.975))
  }

  list(media, quantiles)

}
```

2.5 Transition probability from refracture to death, $p_{RD}(s,t \mid t_{FR})$

```
p23.func <- function(s,t,t12,x){

  media<-numeric(length(t))
  quantiles<- matrix(nrow=2,ncol=length(t))

  x[2]<- x[2]-mean(edad_i)

  #for each given time we calculate mean and credibility interval
  for(i in 1:length(t)){
    time.diff <- (t[i]-t12)^alpha23-(s-t12)^alpha23
    aux<- 1-exp(-lambda23*time.diff*exp(x[1]*beta123+x[2]*beta223))
    #aux contains the posterior distribution
    media[i]<-mean(aux)
    quantiles[,i] <- quantile(aux, probs=c(0.025,0.975))
  }

  list(media, quantiles)
}
```

2.6 Transition probability from fracture to death, $p_{FD}(s,t)$

```
p13.func<- function(s,t,x){

  media<- numeric(length(t))
  quantiles<- matrix(nrow=2,ncol=length(t))

  x[2]<- x[2]-mean(edad_i)

  exp12<-exp(x[1]*beta112+x[2]*beta212)
  exp13<-exp(x[1]*beta113+x[2]*beta213)
  exp23<-exp(x[1]*beta123+x[2]*beta223)

  int<- numeric(length(alpha12))
  ext<- lambda12*alpha12*exp12*exp(lambda12*s^alpha12*exp12+
                                   lambda13*s^alpha13*exp13)
```

```
  for(i in 1:length(t)){
    p11<- exp(-lambda12*(t[i]^alpha12-s^alpha12)*exp12-
                lambda13*(t[i]^alpha13-s^alpha13)*exp13)

    for(j in 1:length(alpha12)){

      ff<-function(u){
              u^(alpha12[j]-1)*exp(-lambda12[j]*u^alpha12[j]*exp12[j]-
                                    lambda13[j]*u^alpha13[j]*exp13[j]-
                                    lambda23[j]*(t[i]-u)^alpha23[j]*exp23[j])}
      int[j]<-integrate(ff,s,t[i],rel.tol = 1e-8)[[1]]
    }
    aux<- 1-p11-int*ext #p13= 1-p11-p12
    media[i]<- mean(aux)
    quantiles[,i] <- quantile(aux, probs=c(0.025,0.975))

  }

  list(media,quantiles)

}
```
